# Mitochondrial Dynamics, Mitophagy, and Mitochondria–Endoplasmic Reticulum Contact Sites Crosstalk Under Hypoxia

**DOI:** 10.3389/fcell.2022.848214

**Published:** 2022-02-25

**Authors:** Shuying Wang, Jin Tan, Yuyang Miao, Qiang Zhang

**Affiliations:** Department of Geriatrics, Tianjin Medical University General Hospital, Tianjin Geriatrics Institute, Tianjin, China

**Keywords:** mitochondria, mitochondrial dynamics, mitophagy, mitochondria-endoplasmic reticulum contact sites, hypoxia

## Abstract

Mitochondria are double membrane organelles within eukaryotic cells, which act as cellular power houses, depending on the continuous availability of oxygen. Nevertheless, under hypoxia, metabolic disorders disturb the steady-state of mitochondrial network, which leads to dysfunction of mitochondria, producing a large amount of reactive oxygen species that cause further damage to cells. Compelling evidence suggests that the dysfunction of mitochondria under hypoxia is linked to a wide spectrum of human diseases, including obstructive sleep apnea, diabetes, cancer and cardiovascular disorders. The functional dichotomy of mitochondria instructs the necessity of a quality-control mechanism to ensure a requisite number of functional mitochondria that are present to fit cell needs. Mitochondrial dynamics plays a central role in monitoring the condition of mitochondrial quality. The fission–fusion cycle is regulated to attain a dynamic equilibrium under normal conditions, however, it is disrupted under hypoxia, resulting in mitochondrial fission and selective removal of impaired mitochondria by mitophagy. Current researches suggest that the molecular machinery underlying these well-orchestrated processes are coordinated at mitochondria–endoplasmic reticulum contact sites. Here, we establish a holistic understanding of how mitochondrial dynamics and mitophagy are regulated at mitochondria–endoplasmic reticulum contact sites under hypoxia.

## Introduction

In most eukaryotes, oxygen is necessary for a multitude of cellular processes. An acute hypoxia stress is directly associated with cell death, while cells exposed to chronic hypoxia always suffer from metabolic disorders. Mitochondria are bilayer-membrane-bound organelles within eukaryotic cells, which serve as the main energy-producing organelles. They are highly sensitive to the hypoxia stress and respond dynamically under hypoxia. Although these organelles are first and foremost healthy producers of adenosine triphosphate, they can also transform into toxic reactive oxygen species (ROS) generators under hypoxia, contributing to cell damage (Thomas and Ashcroft 2019). Increasing evidence shows that abnormal metabolic cues induced in hypoxic cells lead to the disruption of mitochondrial dynamic balance, which is considered to be a prominent mechanism of mitochondrial dysfunction, resulting in a series of intracellular signaling cascades and cell apoptosis. Correspondingly, alterations in this dynamic balance play a crucial role in the progression of diverse diseases. Recent studies have shown that mitochondrial abnormalities may be one of the pathological mechanisms of the obstructive sleep apnea-related cardiac injury (Han et al., 2018; Zhang et al., 2021), while maintaining the integrity of mitochondria allows the survival of cardiomyocytes under hypoxic conditions (Pang et al., 2021). Moreover, the disorder in mitochondrial dynamic balance serves as an underlying mechanism in hypoxia-induced kidney damages, which may provide a novel therapeutic target for diabetic nephropathy (Jiang et al., 2020). Furthermore, the disturbance of mitochondrial dynamic balance leads to the loss of functional pancreatic beta cell mass under hypoxia, which is one of the main pathological substrates of diabetes (Zhang et al., 2018). In addition, since hypoxia is one of the important hallmarks of tumor microenvironment, numerous studies have elucidated that the dynamic imbalance of mitochondria plays critical roles in the progression of neoplastic diseases (Francis et al., 2018; Lin et al., 2020). These findings indicate that the dynamic balance of mitochondria is one of the important factors in maintaining the stability of mitochondrial network, which ensures a requisite number of functional mitochondria presented to meet different needs of cells.

Mitochondrial dynamics is one of the fundamental ways in which cells achieve such a control over the population and function of their mitochondria under stress conditions, allowing mitochondria to respond and accommodate immediately to cellular stress (e.g., hypoxia). It contains two opposing processes: mitochondrial fission and fusion. Mitochondrial fission promotes the segregation of injured mitochondria and enables the selective elimination of dysfunctional mitochondria. Relatively, mitochondrial fusion is a complementary route that aids in the stress alleviation by diluting components of partially impaired mitochondria, and consequently prevents excessive clearance of mitochondria (Youle and van der Bliek 2012; Chan 2020; Dorn 2020). Under normal circumstances, the fusion-fission cycle is orchestrated to attain a dynamic equilibrium required to sustain both the structure and function of mitochondria. The dynamic equilibrium is important for the fitness of cells, especially for energy-intensive cells that are particularly vulnerable to mitochondrial defects, such as neurons, cardiomyocytes and tumor cells. Nevertheless, the equilibration is disrupted and swung to a new one when the content of oxygen reduces, acquiring a highly fragmented mitochondria network, which affects a wide range of cellular functions and can be observed in many human diseases (Chan 2020).

Another quality-control aspect of mitochondria is to dispose those impaired one by autophagy, characterized by the engulfment of unwanted cellular materials, such as the protein aggregates, damaged organelles or intracellular pathogens, through double-membrane encapsulated structures known as autophagosomes, delivering their cargo to the lysosome or vacuole for degradation and recycling (Pohl and Dikic 2019). The engulfment of mitochondria in lysosomes was first described about 40 years ago, before the term of mitophagy was introduced, which refers to the selective removal of injured or ruptured mitochondria by the autophagy apparatus (Tan et al., 2016; Gustafsson and Dorn 2019). Meanwhile, it also represents a critical adaptive mechanism that prevents elevated ROS levels and cellular demises under hypoxic conditions.

A great number of studies have shown that the endoplasmic reticulum (ER) and mitochondria exhibit extensive contacts and intimate dynamic connections. These specific contact spots, known as Mitochondria–Endoplasmic Reticulum Contact Sites (MERCSs), are also essentially required for implementing mitochondrial functions. Mechanistically, current researches provide evidence for a complicated link between mitochondria dynamics and mitophagy, which are integral to the metabolic adjustment of cells exposed to hypoxia. Recent studies have shed light on the role of MERCSs, serve as an important signaling hub, in regulating mitochondrial dynamics and mitophagy under hypoxia. Moreover, machineries of these reactions have been demonstrated to assemble at MERCSs to regulate the mitochondrial network in response to hypoxia stress. In this review, we focus on the current understanding of mechanisms that modulate each process, and establish a holistic understanding of how mitochondrial dynamics and mitophagy are regulated at MERCSs under hypoxia.

## Mitochondrial Dynamics and Its Equilibrium

### Mitochondrial Fission and Fusion Machineries

**FIGURE 1:**
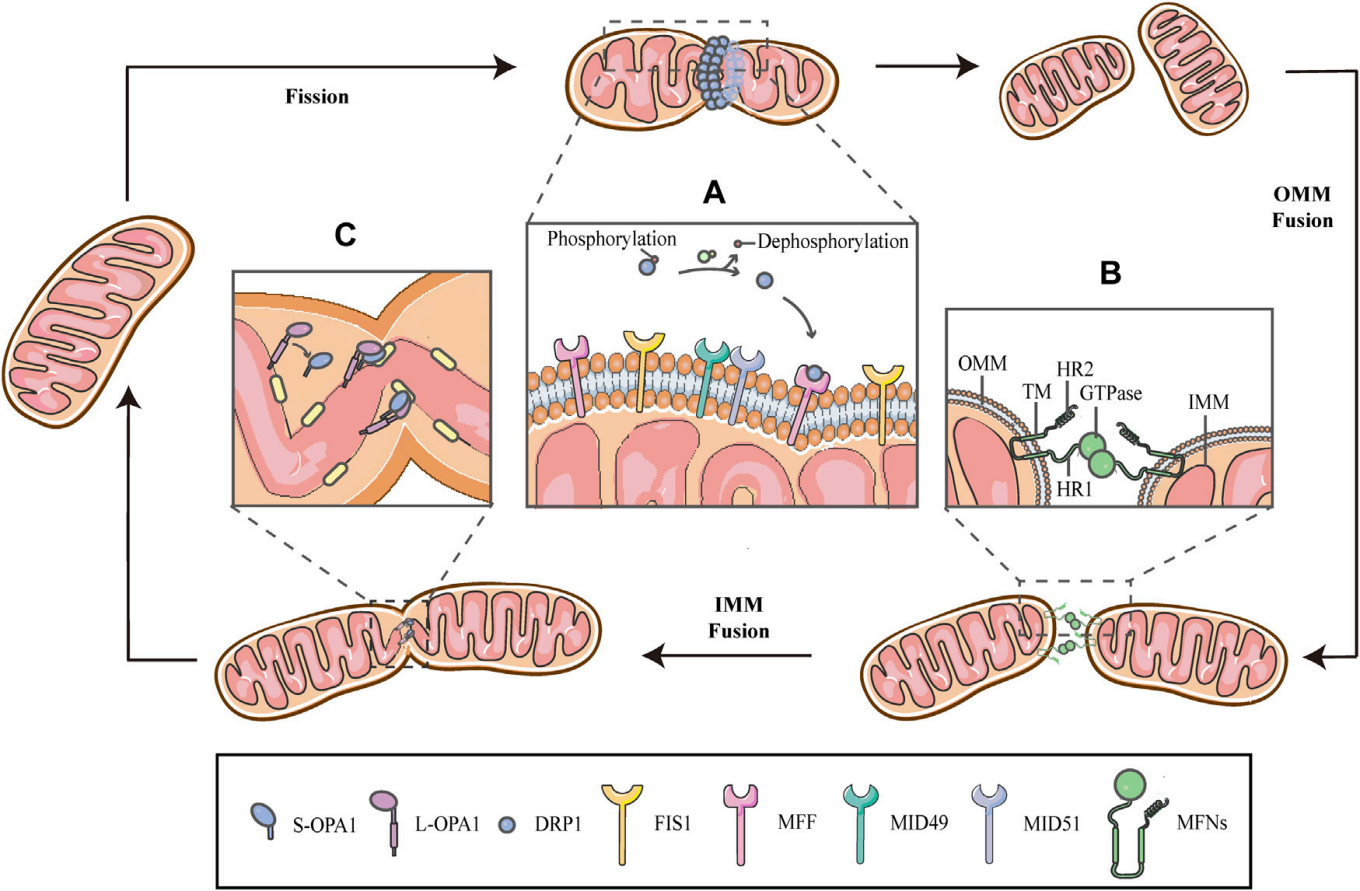
Schematic diagrams of mitochondrial fission-fusion cycles. Mitochondria constantly undergo fission and fusion cycles to maintain a characteristic morphology in dynamic balance, allowing the mitochondrial network to adapt to cellular demands and immediately respond to imposed stresses. **(A)** Mitochondrial fission is mainly executed by DRP1, which is phosphorylated on Ser637, keeping it in the cytosol. When fission occurs, the cytosolic DRP1 is dephosphorylated by calcineurin and recruited to the OMM through interactions with the corresponding receptors, including MFF, FIS1 and MiD49/51, and then DRP1 self-assembles to form a ringlike structure and induces GTP hydrolysis to mediate membrane constriction. **(B)** Mitochondrial fusion begins with the OMM fusion, which is controlled by the MFNs. The MFNs is inserted into the OMM via two TM domains, separated by a short loop, exposing the HR2 domain and the GTPase with HR1 domain, both of which face the cytoplasm. The specific trans-structure underlies MFNs-mediated OMM fusion. **(C)** Following OMM fusion, the interaction between L-OPA1 and CL drives the fusion of IMM. The interaction is enhanced in the presence of S-OPA1, which is generated by proteolytic cleavage of L-OPA1.

Mitochondria are remarkably motile organelles that can respond to changing cellular environments by rearranging their morphology in a reversible and cyclic manner, through division and fusion of the inner and outer mitochondrial membranes (IMM and OMM), thus presenting a short rod-like shape or interconnected pattern, respectively. This dynamic behavior is termed as mitochondrial dynamics that encompasses fusion and fission of mitochondrial membranes (Figure 1) (Zhan et al., 2013). Previous studies have shown that the fission-fusion cycle is elaborately regulated by a highly conserved mechanism, involving a collection of proteins that are recognized as the dynamin family of GTPases, attached to or located on the IMM and OMM. Fission proteins essentially include dynamin-related protein 1 (DRP1) and dynamin-2 (DNM2), whereas fusion is specifically carried out by mitofusins 1/2 (MFN1/2) and optic atrophy 1 (OPA1) (Youle and van der Bliek 2012; Lee et al., 2016).

Mitochondrial fission is primarily executed by DRP1, which translocates from the cytosol to the outer surface of the mitochondria. Before DRP1 is recruited, it is regulated by a remarkable number of post-translational modifications, including phosphorylation/dephosphorylation (Ko et al., 2016; Edwards et al., 2020), ubiquitination (Nakamura et al., 2006), SUMOylation (Figueroa-Romero et al., 2009), S-nitrosylation (Cho et al., 2009) and glycosylation, in a cell-specific manner (Gawlowski et al., 2012). Phosphorylation-dephosphorylation events bidirectionally regulate the activity of DRP1; phosphorylation of DRP1 on serine637 (Ser637) keep it in the cytosol, while dephosphorylation of the same residue by calcineurin activates DRP1 and drives DRP1-mitochondrial association (Figure 1A) (Flippo et al., 2018; Chen et al., 2020; Edwards et al., 2020; Flippo et al., 2020). This interaction is facilitated by corresponding receptors on the OMM, including mitochondrial fission factor (MFF), fission protein 1(FIS1) and mitochondrial dynamics proteins of 49/51 kDa (MiD49/51) (Figure 1A) (Kalia et al., 2018; Yang and Svitkina 2019). Subsequently, DRP1 oligomerizes to form a helical structure to drive the constriction of the mitochondrial tube (Figure 1) (Kalia et al., 2018). There have, however, been hinted in the literature that the DRP1 spiral is not sufficient for the initiation of mitochondrial constrictions. Structural biology studies have revealed that the inner diameter of this multimeric ring (<200 nm) is smaller than the average diameter of a mitochondrion (∼1 μm), implying the requirement for pre-constriction events that precede DRP1 ring formation (Yang and Svitkina 2019; Cho and Sun 2020). The IMM constriction is thought to be a spontaneous and repetitive Constriction of Mitochondrial Inner Compartments (CoMIC), as a priming event for fission, which occurs predominantly on the IMM, independent of the OMM. The regulation of CoMIC will be specified below. Moreover, transient assembly of actin filaments at the OMM has been observed prior to DRP1 recruitment, which contributes to DRP1-dependent OMM fission (Li et al., 2015; Moore et al., 2016a). In addition, other proteins involved in the regulation of actin cytoskeleton, such as Arp2/3 (Li et al., 2015), cofilin (Li et al., 2015; Rehklau et al., 2017; Li et al., 2018), cortactin (Li et al., 2015) and Septin 2 (Pagliuso et al., 2016), have been demonstrated to be involved in the regulation of mitochondrial fission.

**FIGURE 2:**
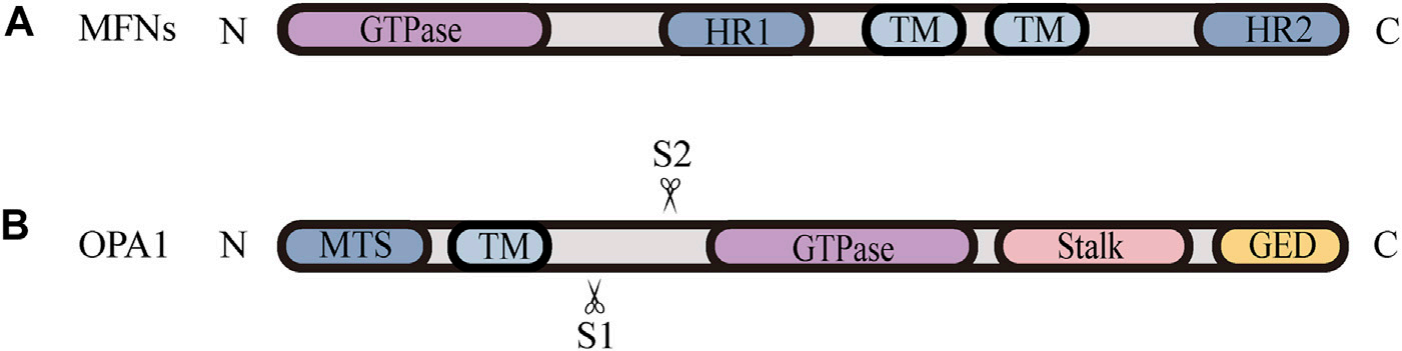
Schematic diagrams of the structural elements of fusion proteins. **(A)** The MFNs contains two TM domains, separated by a short loop, exposing the N-terminal region harbouring the GTPase and HR1 domain, and the C-terminal containing the HR2 domain. **(B)** The structure of unprocessed OPA1 includes the N-terminal MTS, TM domain, GTPase domain, middle stalk region, and the C-terminus containing the GED. OPA1 can be proteolytically processed by YME1L or OMA1 at S1 or S2, respectively, leading to the accumulation of L‐OPA1 or S‐OPA1.

Mitochondrial fusion is a process that two mitochondria collide end-to-end, in a typical fusion reaction, and eventually merge into one, accompanied by the mixing of their contents. It can also occur end-to-side, or within a single mitochondrion to form ringlike morphologies (Chan 2020). It is proceeding through two consecutive processes, the OMM fusion and followed IMM fusion. MFN1/2 and OPA1 are different members of the large GTPase of the dynamin superfamily, which control the process of mitochondrial fusion. The MFNs is inserted into the OMM via two transmembrane (TM) domains, separated by a short loop, exposing the N-terminal region harbouring the GTPase and the coil-coil heptad repeat 1 (HR1) domain, and the C-terminal containing the HR2 domain, both of which face the cytosol (Figures 1B, 2A) (Tilokani et al., 2018; Brandt et al., 2016). The MFNs is a core component of mitochondrial fusion, which functions as a tether between fused mitochondria, leading to membrane clustering, and the specific trans-structure underlies MFNs-mediated OMM fusion (Figure 1B). Previously, the functional overlap was demonstrated between MFN1 and MFN2 in mitochondrial fusion, and structural and functional similarities between them enabled partial compensation for the loss of each other (Detmer and Chan 2007). Furthermore, MFN1 and MFN2 have been reported to share a high degree of homology and sequence similarity (De Vecchis et al., 2019). Following the OMM fusion, the IMM fusion is governed by OPA1 (Figure 1C). OPA1 can cooperate with MFNs to regulate fusion of the inner- and outer- membranes. Although OMM fusion was shown in OPA1-deficient cells, the abortive IMM fusion, caused by the loss of OPA1, resulted in the fragmentation of mitochondrial network (Mishra et al., 2014; Ge et al., 2020). The structure of OPA1 includes the N-terminal mitochondrial transit sequence (MTS), TM domain, GTPase domain, middle stalk region, and GTPase effector domain (GED) at the C-terminus (Figure 2B) (Faelber et al., 2019; Ge et al., 2020). Actually, OPA1 is a unique member of the dynamin superfamily, because it can be processed into two forms via alternative splicing at two distinct sites, S1 and S2 in a region between the N-terminal to the GTPase domain. Two IMM bound proteases, the ATP-dependent zinc metalloprotease YME1L1 and the ATP-independent metalloendopeptidase OMA1, are responsible for the proteolysis of OPA1, after which the long form of OPA1 (L-OPA1) is proteolytically cleaved is proteolytically cleaved (Anand et al., 2014; Lee et al., 2020). L-OPA1 and cardiolipin (CL, an IMM-specific lipid) interact on opposite sides of two mitochondrial membranes and tether the IMM, in addition, S-OPA1 can enhance the interaction, and then OPA1-dependent GTP hydrolysis ensures the fusion of these two mitochondria (Figure 1C) (Ban et al., 2017).

### The Modulation of Mitochondrial Dynamics Equilibrium

Despite the fact that mitochondria constantly undergo series of dynamic movements that occur along the cytoskeleton, they are somehow able to maintain a characteristic morphology under the dynamic balance, which is fundamental for their diverse and complex activities. Rather than simply controlling mitochondrial size, mitochondrial dynamics involves biological events with series of pathophysiological effects, moreover, breaking the equilibration of mitochondrial dynamics under stress conditions will cause mitochondrial dysfunction, mediating a large variety of diseases. Indeed, the constitutive fission-fusion cycle has been fine-tuned and operates in appropriate equilibrium, which is a key determinant of the overall mitochondrial network structure, supporting normal mitochondrial function. There is also compelling evidence that the proper balance of the repetitive cycle of fission and fusion seems to be more significant than the absolute level of each process. As shown by Song and colleagues in cultured fibroblasts and in vivo mouse hearts, mitochondrial fission performs a critical triage function, isolating normal and damaged components into daughter organelles that can be selectively eliminated by mitophagy, while mitochondrial biogenesis and fusion consistently function to repair, regenerate, and reintroduce healthy daughter mitochondria into the mitochondrial pool. When the fission-fusion cycle remains intact, impaired organelles are properly targeted for removal, and healthy organelles are renewed (Song et al., 2015). Moreover, Song and colleagues have demonstrated that dynamism-defective mice live much longer than mice with fission defects or fusion defects alone, because the forced mitochondrial dynamism can temporarily mitigate the dynamic imbalance between fission and fusion. However, the abrogation of both fission and fusion impairs mitophagy, leading to massive and progressive accumulation of mitochondria and distorted sarcomere structures in cardiomyocytes, and over time, these adynamism mice perform mitochondrial senescence and heart failure (Song et al., 2017). The study also suggests that low levels of mitochondrial fission-fusion events are compatible with high levels of cellular function, as long as these two processes are appropriately balanced. Collectively, mitochondrial fission and fusion are critical to cellular health, moreover, the proper balance of mitochondrial fusion and fission is more important than the individual integrity of either process. Both hyper-fragmented and hyper-fused mitochondrial collectives are inextricably linked to metabolic disruption and negative effects on cellular fitness, which can lead to a wide range of diseases, thus highlighting the importance of mitochondrial dynamic balance (Mishra and Chan 2016). In addition, recent studies provide cumulative evidence that mitochondrial function is apparently restored by rebalance of mitochondrial dynamics. For example, Guo et al. (2020) have reported that rebalancing mitochondrial dynamics can ameliorate cardiac dysfunction in type 2 diabetic mice (Guo et al., 2020).

Nevertheless, the knowledge regarding how fission and fusion machineries occur in a coordinated manner and retain a proper equilibrium is vague. There is accumulating evidence that the proteolytic cleavage of OPA1 is a major regulatory step in balance mitochondrial fusion and fission. The processing of OPA1 is regulated by two mitochondrial inner membrane proteases, YME1L and OMA1, moreover, both of them have been proven to be stress-sensitive mitochondrial proteases, which can be stimulated to respond to cellular injury (Rainbolt et al., 2016). Remarkably, different cellular stresses modulate OPA1 through differential processing by YME1L or OMA1 at S1 or S2, respectively, leading to the accumulation of L-OPA1 or S-OPA1 Figure 2B. YME1L-dependent OPA1 processing promotes the production of L -OPA1, leading to tubular mitochondrial morphology, while OMA1-dependent OPA1 processing generates more S-OPA1, which inhibits fusion and induces mitochondrial fragmentation (Rainbolt et al., 2016). Knockdown of YME1L can activate OMA1, increasing the conversion of L-OPA1 into S-OPA1, thereby promoting fission of mitochondria (Anand et al., 2014). Additionally, Wu et al. (2018) have demonstrated that the imbalance in YME1L and OMA1 activities, afforded by their abnormal degradation, is associated with OPA1 dysfunction, which induces the imbalance in mitochondrial dynamics (Wu et al., 2018). For example, *in vivo* models of neonatal hypoxic-ischemic (HI) injury show an accumulation of S-OPA1 and an alteration in mitochondria morphology, which are attributed to the aberrant cleavage of OPA1 (Baburamani et al., 2015). Baburamani et al. (2015) also found that the level of YME1L was reduced while the activated OMA1 was stabilized in these HI models, implying that the imbalance in stress-dependent regulation of YME1L and OMA1 leads to OPA1 dysfunction, contributing to the disruption of mitochondrial dynamic balance and pathologic mitochondria dysfunction (Baburamani et al., 2015). Consistent with these findings, it is observed that the deletion of OMA1 can rescue mitochondrial damage induced by YME1L depletion *in vitro* and *in vivo* (Baker et al., 2014; Wai et al., 2015b). Accordingly, the proper balance of these two OPA1 isoforms, which is regulated by OMA1- and YME1L-dependent OPA1 differential processing, is thought to be a key determinant in the coordination of mitochondrial fission and fusion under various physiological conditions.

In cells under normal physiological conditions, the constitutive cleavage of S1 and S2 sites establishes a near equimolar balance of two OPA1 isoforms, a combination that is optimal for the physiological level of mitochondrial fusion (Chan 2020; Ge et al., 2020). However, the activity of OMA1 is enhanced under stress conditions, such as hypoxia, which leads to the cleavage of L-OPA1 at the S1 site and the accumulation of S-OPA1, which facilitates mitochondrial fission, resulting in fragmentation of the network. And the stimulated fission promotes mitochondrial recycling through mitophagy and supports cell survival. Simultaneously, the activated OMA1 undergoes autocatalytic degradation, allowing the recovery of the mitochondrial dynamic equilibrium and the restoration of mitochondrial morphology upon stress alleviation (Baker et al., 2014; Rainbolt et al., 2016). If the stress persists, nevertheless, unopposed mitochondrial fragmentation can ultimately lead to cell death. The analysis of mouse models revealed that the accelerated OPA1 processing by stress-induced OMA1 activation led to mitochondrial fragmentation in cardiomyocytes and neurons and cell death, while deleting OMA1 or preventing excessive mitochondrial fission, such as DRP1 inhibition, was demonstrated to be cyto-protective in the mouse heart and brain (Wai et al., 2015a; Korwitz et al., 2016).

**FIGURE 3:**
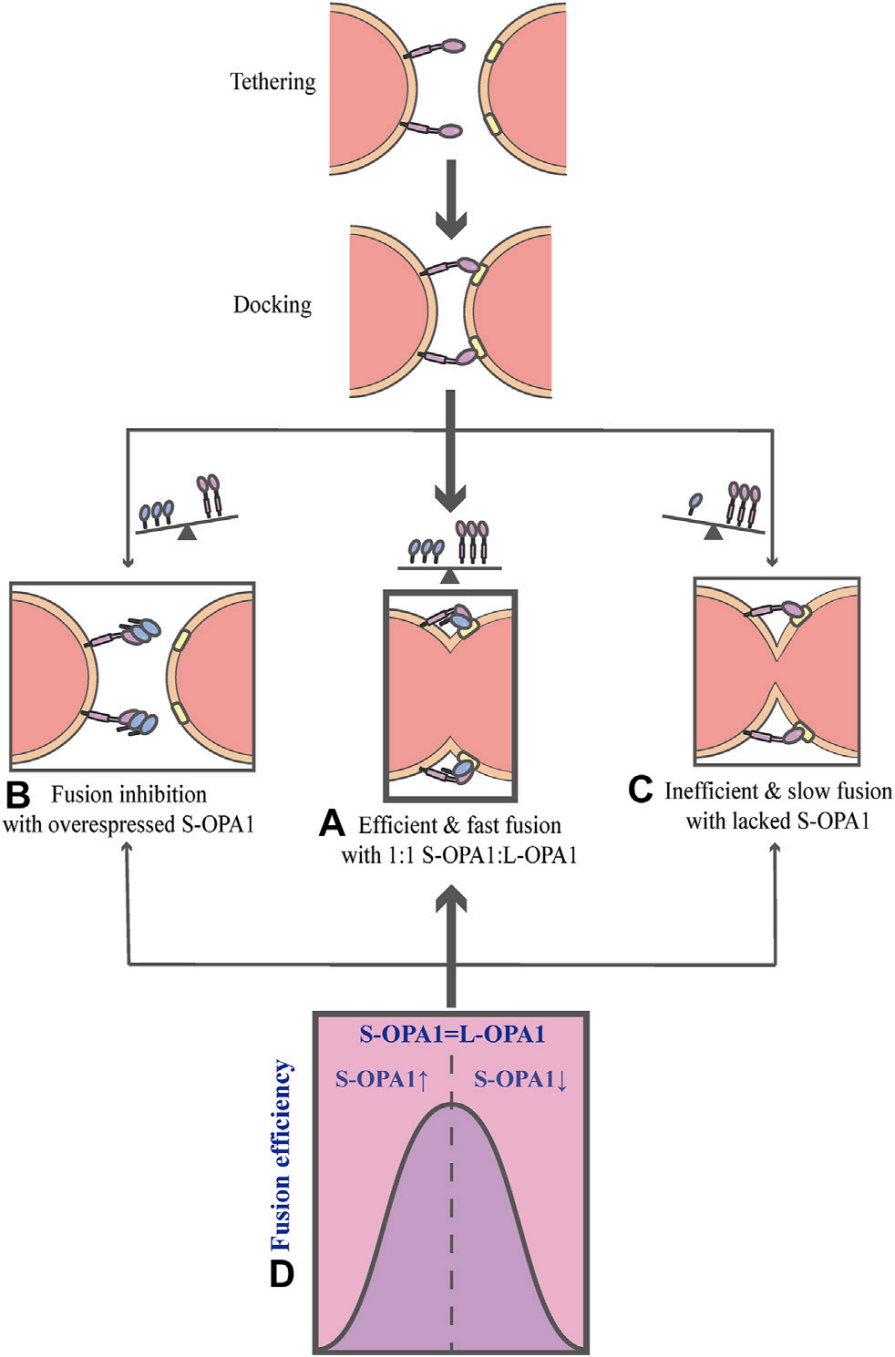
Schematic diagrams of OPA1-dependent IMM fusion. The process of mitochondrial IMM fusion includes several sequential steps: tethering, membrane docking and fusing. **(A)** It exhibits efficient and fast fusion at equimolar levels of S-OPA1 and L-OPA1. **(B)** When S-OPA1 is overexpressed, the activity of fusion is inhibited and the fusion efficiency is reduced. **(C)** When the S-OPA1 level is lower than L-OPA1, slow and inefficient fusion occurs. **(D)** The activity of the IMM fusion peaks at a ratio of 1:1S-OPA1: L-OPA1.

**FIGURE 4:**
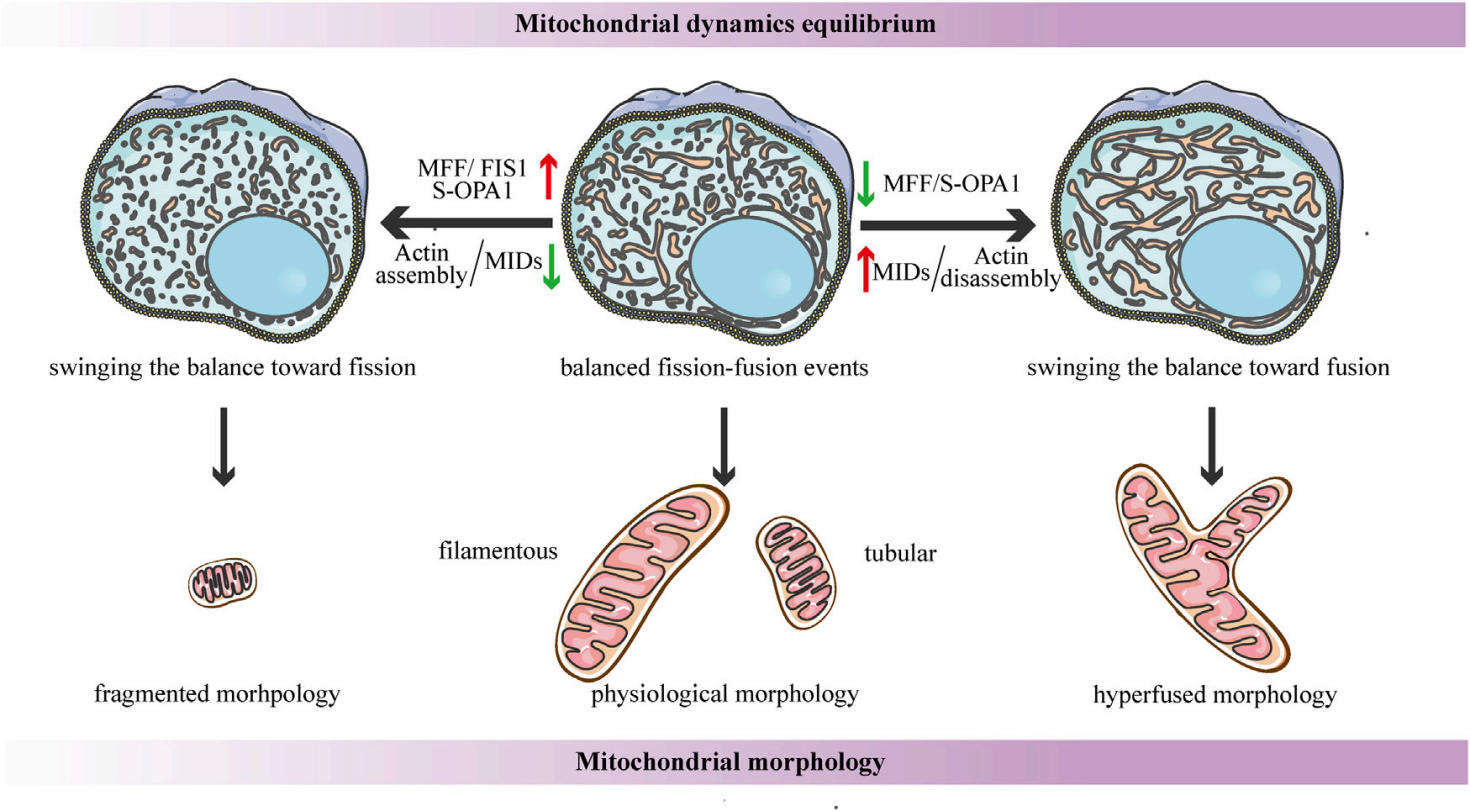
Schematic diagrams of the regulation of mitochondrial dynamics equilibrium and its connection to mitochondrial morphology. Under steady-state conditions, the fission-fusion cycle is constantly maintained in an appropriate equilibrium, bringing about characteristic, filamentous or tubular mitochondrial morphologies. This dynamic balance can be affected by many factors. The dynamic actin cycle plays an important role in the regulation of mitochondrial dynamics equilibrium. Actin assembly induces mitochondrial fission, whereas actin disassembly promotes fusion. Moreover, S-OPA1 and the corresponding receptors of DRP1 are involved in the regulation of mitochondrial dynamics balance. The overexpression of MFF/FIS1/S-OPA1 can tip the balance toward fission, similarly, low-to-moderate levels of MIDs prefer to shift the balance toward fission, leading to a fragmented mitochondrial network. In contrast, the overexpression of MIDs or the low-to-moderate level of S-OPA1, as well as the inhibition of MFF, can swing the dynamic equilibrium in the direction of fusion, resulting in a hyperfused mitochondrial morphology.

As mentioned above, OPA1 can be proteolytically processed into two forms: L-OPA1 and S-OPA1. Previous researches have revealed that L-OPA1 is competent for mitochondrial fusion, but S-OPA1 is not (Lee et al., 2017). A previous study has shown that fusion occurs in the YME1L^−/−^, OMA1^−/−^ background, in which L-OPA1 is expressed alone without being cleaved, whereas the overexpression of S-OPA1 results in a fragmented mitochondrial network (Anand et al., 2014). Moreover, the abolishment of OPA1 cleavage by OPA1 mutation reveals that S-OPA1 is dispensable for fusion and may even involve in fission (Baker et al., 2014; Lee et al., 2017). However, a current research suggests that both L-OPA1 and S-OPA1 can display inherent fusion activity (Ge et al., 2020). Intriguingly, the research uses an *in vitro* reconstitution system to establish a model of the IMM fusion, which includes several sequential steps: tethering, membrane docking and fusing (Figure 3) (Ge et al., 2020). It also describes the key mechanism of how the IMM fusion process is regulated by the ratio of two forms of OPA1 (Ge et al., 2020). In this work, Ge et al. (2020) have found that S-OPA1 can strongly stimulate the activity of L-OPA1-dependent IMM fusion, notably, this activity peaks at a ratio of 1:1S-OPA1: L-OPA1, suggesting that S-OPA1 and L-OPA1 cooperate to facilitate fast and efficient fusion (Figures 3A,D) (Ge et al., 2020). Furthermore, excessive amounts of S-OPA1 inhibit the activity of fusion and reduce fusion efficiency (Figure 3B), whereas, when the level of S-OPA1 is lower than that of L-OPA1, it leads to slow and inefficient fusion (Figures 3C and 4) (Ban et al., 2017; Ge et al., 2020). More importantly, when fusion is impaired, the balance of mitochondrial dynamics is disturbed and shifts to fission. As pointed out by Ge et al. (2020), the activity of fusion is suppressed under conditions of S-OPA1 overexpression, thereby swinging the equilibrium of mitochondrial dynamics toward fission (4) (Ge et al., 2020). Therefore, the adjustment of the ratio of two OPA1 forms may be a potential target for regulating the balance of mitochondrial dynamics and requires further investigation. Other studies strongly suggest that S-OPA1 not only suppresses fusion activity, but is mechanistically linked to mitochondrial fission, eventually tipping the balance toward fission (Anand et al., 2014; Wai et al., 2015b). Ectopic expression of S-OPA1 in DKO cells (cells lacking both YME1L and OMA1) induced fragmentation of the mitochondrial network, moreover, S-OPA1 colocalized with mitochondrial fission sites, as shown by fluorescence microscopy, implying partial colocalization of the S-OPA1 with mitochondrial constriction sites containing punctate with DRP1 or MID49/50 spots (Anand et al., 2014). Furthermore, Cho et al. have demonstrated that S-OPA1 contributes to IMM constriction by promoting the induction/potentiation of CoMIC and preventing IMM-OMM tethering. Accordingly, S-OPA1 plays a stimulatory role in mitochondrial fission and contributes to the coordination of mitochondrial fusion and fission. Taken together, these *in vivo* and *in vitro* data have firmly established OPA1 as a key modulator of mitochondrial dynamics, coordinating fusion and fission of mitochondria.

Another research proposes that the dynamic actin cycle also plays an important role in the regulation of mitochondrial dynamics equilibrium. Using live-cell imaging, they have found that before actin recruitment, mitochondria are typically interconnected and tubular, while focal assembly of actin results in mitochondrial fragmentation and inhibits fusion ability (Moore et al., 2016a). The polymerization of actin around a distinct mitochondrial subpopulation is transient, and it quickly disassembles, leading to local recovery of the tubular mitochondrial network, then reassembles around another subpopulation, cycling quickly and efficiently through all cellular mitochondria (Moore et al., 2016a). Taken together, these findings suggest that the cycle of actin polymerization/depolymerization on mitochondrial subpopulations can modulate the fission-fusion balance (Figure 4). In addition, it has been shown that corresponding receptors of DRP1 may be involved in the regulation of mitochondrial dynamics balance. A recent study proposes a novel function for human FIS1 in regulating mitochondrial dynamics. It has been shown that the overexpressed-FIS1 inhibits the GTPase activity of MFN1, MFN2, and OPA1, blocking fusion activity, and thus independently promoting fission in the absence of DRP1 (Figure 4) (Yu et al., 2019). The contribution of MFF to mitochondrial fission seems to be straightforward in the sense that DRP1 recruitment is hindered due to MFF deletion, whereas the overexpressed-MFF enhances mitochondrial fragmentation (Figure 4) (Chen et al., 2015; Wu et al., 2021). Conversely, the function of MID49/51 is more complicated, and they appear to have opposite effects on mitochondrial morphology according to their expression levels. Low-to- moderate levels of MIDs promote mitochondrial fission, while the overexpressed-MIDs results in mitochondrial elongation most likely by inhibiting fission (Figure 4) (Yu et al., 2017). This divergence may be due to the inverse regulation of the GTPase activity of DRP1--the MIDs suppresses while MFF stimulates the GTPase activity (Osellame et al., 2016).

## Mitophagy Is Coupled To the Disturbance of Mitochondrial Dynamics Under Hypoxia

### The Equilibrium of Mitochondrial Dynamics Is Disturbed and Swung Toward Fission Under Hypoxia

Under steady-state conditions, the fusion-fission cycle is constantly maintained to an appropriate equilibrium, which determines the size, number and form of mitochondria. However, when the cellular environment changes or when cells are subjected to appropriate stimuli, such as starvation, UV radiation and hypoxic insults, the dynamic balance of mitochondria will be disrupted, resulting in mitochondrial abnormalities. The blocking of fusion activity tilts the balance in favor of fission, leading to small punctate or fragmented mitochondria. Conversely, the balance is swung toward fusion when fission is blocked, preserving dramatically elongated or hyperconnected mitochondria (Spurlock et al., 2020). In fact, alterations in mitochondrial dynamics play a key role in response to fluctuating physiological cues within the cell, whereas severe cellular stress can break the equilibrium and typically drive the cycle toward either extreme. In addition, it has been shown that the efficiency of ATP productions is increased and the exchange of matrix contents is favored in fused mitochondria, whereas fragmented mitochondria may generate more ROS and can be efficiently removed by mitophagy (Giacomello et al., 2020).

As major consumers of oxygen in cells, mitochondria are extremely sensitive to the fluctuation of oxygen concentration in the atmosphere, which makes them prime targets for hypoxia damages. Moreover, the alteration in mitochondrial dynamics is also closely bound up with the availability of oxygen. Under normoxia, mitochondria form tubular networks favoring ATP production, however, under hypoxic conditions, the dynamic balance of the fission-fusion cycle is disturbed and swung toward fission, resulting in a hyper-fragmented mitochondrial network. This notion has been validated in numerous studies by through different cell or animal models. Increased mitochondrial fission was shown in mouse pancreatic beta cells (Zhang et al., 2018) and neurons (Baburamani et al., 2015; Flippo et al., 2018), as well as in cultured human pulmonary arterial smooth muscle cell (Chen et al., 2019) upon hypoxic treatment. Similar phenotype was observed in mouse primary cardiomyocytes (Han et al., 2018; Nishimura et al., 2018; Xin et al., 2020). Using immunofluorescence, Xin et al. (2020) have shown that mitochondria are characteristic, spindle-shaped under normoxia, whereas their forms become small and round under hypoxia (Xin et al., 2020). Emerging studies have revealed underlying mechanisms by which hypoxia regulates mitochondrial dynamics. Some of the studies have shown that mitochondria respond to hypoxia by regulating the expression of mitochondrial dynamics proteins. Gene expression analysis in cardiomyocytes showed that mitochondrial fission-related genes DRP1, MFF, and FIS1 were upregulated, while mitochondrial fusion-related genes MFN1 and MFN2 were downregulated under hypoxia (Xin et al., 2020). Analogously, the expression of OPA1 was downregulated in hypoxia-treated cardiomyocytes (Xin and Lu 2020). Several other studies reported that during hypoxia-ischemia insults, the major alteration of OPA1 in mouse neurons and human cardiomyocyte cell lines was its rapid cleavage to a shorter form, which promoted mitochondrial fission (Baburamani et al., 2015; Sanderson et al., 2015; Pang et al., 2021). Furthermore, several upstream regulatory molecules have been identified to regulate mitochondrial dynamics under hypoxic conditions. It has been found that the ubiquitin ligase Siah2 occupies a key point in the control of mitochondrial fission by regulating the availability of the A-kinase anchoring protein 121 (AKAP121) under hypoxia (Kim et al., 2011; Flippo et al., 2018). AKAP121 is a scaffold protein located on the mitochondrial membrane, which can bind to the regulatory subunit of protein kinase A (PKA), allowing the inhibitory phosphorylation of DRP1 by PKA on Ser637 (Kim et al., 2011; Flippo et al., 2018). Moreover, it can inhibit the DRP1-FIS1 interaction through a PKA-independent mechanism (Kim et al., 2011). Under hypoxia, increased expression and activity of Siah2 limit the availability of AKAP121 with a concomitant effect on DRP1 phosphorylation and DRP1-FIS1 complex formation, leading to mitochondrial fission (Kim et al., 2011; Flippo et al., 2018; Sisalli et al., 2020). In addition, Siah2 can regulate the availability of hypoxia-inducible factor 1α (HIF-1α) by inhibiting the activity of prolyl hydroxylases (PHD) under hypoxic conditions (Nakayama et al., 2004). HIF-1α is a DNA binding transcription factor that is stabilized and activated under hypoxia, promoting the upregulation of genes that control cellular adaptive responses (Fuhrmann and Brune 2017). There are several studies focus on the role of HIF-1α in the induction mitochondrial fission under hypoxic conditions. It has been shown that HIF-1α directly regulates DRP1 expression by binding to a hypoxia-responsive element within the promoter of the DRP1 target gene (Kuo et al., 2017; Chen et al., 2019). Another research has found that HIF-1α can also increase the transcriptional expression of MFF under hypoxia, thus enhancing hypoxia-induced mitochondrial fission (Wu et al., 2021). Under hypoxia, the increase of mitochondrial fission allows forming smaller and more discrete mitochondria, promoting mitophagy to remove damaged mitochondria for quality control and cell apoptosis during severe stress (Youle and van der Bliek 2012). That is, the enhanced mitochondrial fission under hypoxia may be a rescue response to hypoxia-induced mitochondrial damage. In addition, there are many other factors that control mitochondrial dynamics under hypoxia, forming a complex regulatory network. Several studies have reported that HIF-1α upregulates the expression of target genes, such as haem oxygenase-1 (HO-1), hypoxia-induced gene domain protein-1A (HIGD-1A) and HIGD-1B, inhibiting hypoxia-induced mitochondrial fragmentation (Fuhrmann and Brune 2017; Han et al., 2018; Jiang et al., 2020; Pang et al., 2021). It is noteworthy that mitochondria are constantly undergoing fission and fusion, and that these two events do not occur as independent processes, however, in general, mitochondrial fission is more intense than fusion under hypoxic conditions.

As described above, DRP1 is at the core of regulating mitochondrial fission, and several mitochondrial receptors, including MFF, FIS1, and MID49/51, are involved in the recruitment of DRP1 during mitochondrial fission. However, recent studies have revealed that these regular binding receptors may primarily be involved in normal mitochondrial dynamics or in other stressful conditions, but play a lesser role in mitochondrial fission under hypoxic conditions (Chen et al., 2016; Wu et al., 2016a; Wu et al., 2016b). Moreover, FUN14 domain-containing 1 (FUNDC1), one of the mitochondrial outer membrane proteins, has been reported to be a new DRP1 receptor that is specifically required for mitochondrial fragmentation in response to hypoxia (Wu et al., 2016a; Wu et al., 2016b; Chen et al., 2016; van der Bliek 2016). As shown in immunogold electron micrographs, DRP1 sporadically localized with mitochondria to facilitate regular mitochondrial fission under normoxia, and the working group also found that FUNDC1 could interact with DRP1, which markedly localized with mitochondria in Hela cells under hypoxia, accompanied by an increasing number of ruptured mitochondria (Wu et al., 2016b). Furthermore, when FUNDC1 was silenced in hypoxic cells, DRP1 translocation to mitochondria was drastically reduced and mitochondrial elongation was observed, whereas knockdown of MFF, FIS1, or MID49/51 in cells did not affect the translocation of DRP1 during hypoxia, and still remained a large percentage of fragmented mitochondria in the cell (Wu et al., 2016b). Collectively, FUNDC1 specifically mediates DRP1 recruitment and facilitates DRP1-dependent mitochondrial fission under hypoxic conditions.

### FUNDC1-Mediated Mitophagy Is Triggered by Enhanced Fission Under Hypoxia

In case of persistent hypoxia, enhanced fission results in a fragmented network, which helps to separate dysfunctional mitochondria from the network, allowing their selective elimination by mitophagy. Since the average diameter of a mitochondrion is much larger than that of an autophagosome, it is necessary for a damage mitochondrion to be fragmented by fission into appropriate sizes, which can be engulfed by autophagosomes and trafficked to the lysosome for degradation during mitophagy, leaving a functional mitochondrial network to promote cellular recovery (Thornton et al., 2018). Lin et al. (2020) found that mitophagy was induced by increasing DRP1-mediated mitochondrial fission in hepatocellular carcinoma cells that survived hypoxia, in contrast, inhibiting fission by blocking DRP1 significantly suppressed hypoxia-induced mitophagy (Lin et al., 2020). Obviously, mitochondrial fragmentation due to imbalanced fission and fusion during hypoxia serves as a prerequisite for mitophagy. An increasing number of studies have focused on the mechanistic underpinning of how mitochondrial fission-fusion events are orchestrated with mitophagy under hypoxia.

Previous studies have shown that the regulatory pathways of mitophagy can be classified into ubiquitin-dependent and receptor-dependent pathways, according to the mode of action and the proteins involved (Tan et al., 2016). The ubiquitin-dependent mitophagy is regulated by the phosphatase and tensin homologue (PTEN)-induced putative kinase 1 (PINK1)–Parkin pathway (Palikaras et al., 2018). Moreover, mitochondrial proteins, including NIP3-like protein X (NIX), BCL2 interacting protein 3 (BNIP3), and FUNDC1, serve as mitophagy receptors that directly participate in mitophagy (Palikaras et al., 2018). It is known that mitophagy occurs through the receptor-dependent pathway during hypoxia (Liu et al., 2012). As one of the mitophagy receptors, FUNDC1 has been shown to play important roles in hypoxia-induced mitophagy (Liu et al., 2012). Recently, compelling evidence has proposed that FUNDC1 is not only a receptor that mediates mitophagy, but also a DRP1 receptor that can recruit DRP1 from the cytoplasm to the OMM, coupling mitochondrial dynamics to mitophagy under hypoxia (Chen et al., 2016; Wu et al., 2016b). Interestingly, FUNDC1 has also been demonstrated to be able to directly interact with both L- and S-OPA1, and the interaction suppresses its activity, inhibiting FUNDC-DRP1 binding and FUNDC1-mediated mitophagy (Chen et al., 2016). Chen et al. (2016) found that OPA1 interacted with FUNDC1 via its lysine 70 (K70) residue, and that FUNDC1K70A (mutation of K70 to arginine) abolished the interaction, consistent with knockdown of OPA1, facilitating mitochondrial fission and mitophagy (Chen et al., 2016). More importantly, they reveal that how FUNDC1 interacts with DRP1 and OPA1 to collaboratively regulate mitochondrial fission-fusion events and mitophagy. Previous studies have shown that FUNDC1 is phosphorylated by casein kinase 2 (CK2) at Ser13 and by SRC tyrosine kinase at tyrosine18(Tyr18) under non-stress conditions, whereas the dephosphorylation of FUNDC1 at Ser13 by PGAM5 phosphatase is important for the initiation of mitophagy in response to hypoxia (Liu et al., 2012; Chen et al., 2014). Furthermore, the phosphorylation/dephosphorylation of FUNDC1 has been shown to play critical roles in the interaction with DRP1 and OPA1 to regulate mitochondrial dynamic balance, consistent with the finding that the phosphorylation status of FUNDC1 regulates mitophagy (Liu et al., 2012; Chen et al., 2016). In conclusion, FUNDC1 is dephosphorylated and activated during hypoxia, dissociating and releasing OPA1, while enhancing FUNDC1-DRP1 binding affinity and recruiting DRP1 to the mitochondrial surface, thereby swinging the mitochondrial dynamics equilibrium toward fission and triggering FUNDC1-mediated mitophagy. In addition, the released OPA1 is converted into S-OPA1 by OMA1 under hypoxia, ultimately, the excessive accumulation of S-OPA1 brings about the inhibition of fusion activity, thus altering the dynamic balance in favor of mitochondrial fission, resulting in small round or short spheres mitochondria (Ban et al., 2017; Ge et al., 2020; Pang et al., 2021).

## Mitochondrial Fission and Fusion Machineries Converge at MERCSs

### MERCSs and Its Intracellular Functions

Mitochondria and the ER are interconnected organelles that exhibit extensive contact sites between them, referred to as MERCSs. Ultrastructural studies have found that MERCSs are abundant in diverse tissues and cell types, which are critical for the coordination of functions of these two organelles. Indeed, it has been proposed to be involved in biochemical and signaling functions, such as lipid synthesis, Ca^2+^ homeostasis, and the control of intracellular transports and mitochondrial dynamics (Rowland and Voeltz 2012; Valm et al., 2017; Aoyama-Ishiwatari and Hirabayashi 2021; Lin et al., 2021). The connection and reaction between the ER and mitochondria are greatly dependent on complementary membrane proteins and lipids, which tether the two organelles to the MERCSs. Various molecules, such as lipids, proteins and ions, are able to be transported between the ER and mitochondria, or are specifically recruited to their unique contact sites, exerting their functions (Lin et al., 2021).

Biochemical studies have revealed that lipid synthases are enriched in MERCSs, and an increasing number of studies have focused on the role of MERCSs in intermembrane phospholipid transport (Vance 1990). The complex interplay of different phospholipid synthases requires bidirectional and constant transport of lipids between the two organelles (Rowland and Voeltz 2012; Petrungaro and Kornmann 2019). However, the underlying mechanism of the exchange and factors involved in lipid transport have not been fully uncovered so far. The MERCSs is also crucial for the regulation of intracellular Ca^2+^ signaling. The ER is the major Ca^2+^ reservoir, and the MERCSs provides channels that can transfer high concentrations of Ca^2+^ from the ER to mitochondria (Cremer et al., 2020). Ca^2+^ is released from the ER through the inositol 1,4,5-triphosphate receptor (IP3R) and enters mitochondrial matrix via voltage-dependent anion channels (VDAC) in the OMM (Szabadkai et al., 2006; Wu et al., 2017). Glucose-regulated protein 75 (GRP75) is a scaffold protein that interacts with both IP3R and VDAC1, improving the efficiency of mitochondrial Ca^2+^ uptake (Szabadkai et al., 2006). Once Ca^2+^ is translocated across the OMM, it can traverse the IMM into the matrix via the mitochondrial calcium uniporter (MCU) (Baughman et al., 2011). Ample evidence has revealed that the transport of Ca^2+^ at MERCSs is important for mitochondrial function, mitochondrial fission and activation of apoptosis (Wu et al., 2017; Chakrabarti et al., 2018; Rowland and Voeltz 2012). Moreover, MERCSs have been revealed to define the sites of both mitochondrial fission and fusion, as discussed specifically below. Additionally, the MERCSs also provides a platform for mitophagosome biogenesis during mitophagy (Böckler and Westermann 2014).

### Multiple Constriction Events of the Inner and Outer Mitochondrial Membranes Occur at MERCSs

**FIGURE 5:**
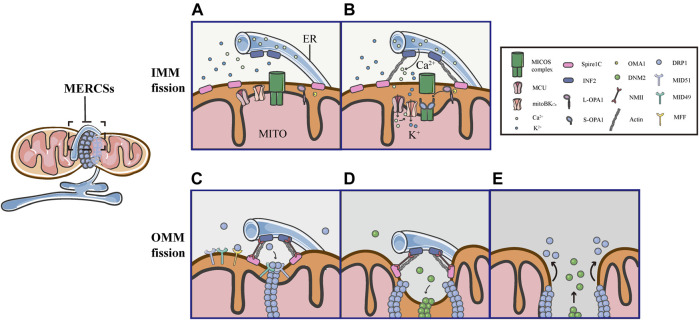
Schematic diagrams of mitochondrial fission at MERCSs. Multiple constriction events of the inner and outer mitochondrial membranes occur at MERCSs. **(A,B)** The pre-constriction events include INF2-mediated actin polymerization and CoMIC, both of which occur at MERCSs, contributing to the IMM constriction. INF2 and spire1C induce actin polymerization that increases ER-mitochondria interactions, thereby stimulating the ER-to-mitochondrial calcium transfer through the MCU. The subsequent increase in intra-mitochondrial Ca^2+^ initiates CoMIC. The elevated mitochondrial Ca^2+^ flux triggers mitoBK_Ca_-mediated mitochondrial bulging and depolarization during CoMIC. OPA1 collaboratively regulates CoMIC through proteolytic processing by stabilized OMA1, resulting in the accumulation of S-Opa1, which disrupts the stability of Mic60-mediated OMM–IMM tethering. **(C)** The actin−NMII-dependent constriction provides the pre-constricted site at the OMM. NMII filaments are present near the constriction site along with actin filaments, both of which ensure the constriction of the actin cable, exerting pressure on mitochondria, bringing about an indentation on the surface of the OMM. DRP1 then assemblies at these pre-constricted sites by binding to its receptors, forming a ringlike structure to further constrict the membrane. **(D,E)** Finally, DNM2 cooperates with DRP1 to drive complete fission before disassembling of the fission machinery.

As mentioned above, the assembly of DRP1 is not sufficient to initiate mitochondrial constriction, suggesting that pre-constriction events are required for efficient mitochondrial division. In addition, using live imaging, it was found that mitochondria were not immediately severed upon the translocation of DRP1 in GFP-DRP1-expressing cells (Ji et al., 2015). Another research showed that the DRP1 ring was insufficient to cut the lipidic structure, an *in vitro* model of a mitochondrial tube, suggesting that additional steps are required for final mitochondrial fission (Basu et al., 2017). Indeed, increasing evidence supports the notion that mitochondria are severed by the protein complex, containing several members, through multiple constriction steps of the OMM and IMM. Remarkably, all of these processes occur at MERCSs, dynamic connection sites between the ER and mitochondria, which act as platforms for the accumulation of the protein complex, coordinating the IMM and OMM constriction (Figure 5).

Several studies have elegantly proposed that actin fibers offer the constriction force required to facilitate the scission of both the inner and outer membranes of mitochondria. As the origin of the pre-constriction event, mitochondria spatially intersect with or are enclosed by the ER, and their contacts are facilitated by the pulling force provided by INF2-mediated actin polymerization (Chakrabarti et al., 2018). Previous studies have shown that the ER-bound protein inverted formin 2 (INF2) is specifically associated with mitochondrial division (Korobova et al., 2013; Manor et al., 2015). Coupling confocal to electron microscopy, two recent works have confirmed that INF2 induces abundant assembly of actin filaments at MERCSs (Moore et al., 2016b; Chakrabarti et al., 2018). ER-associated INF2 and mitochondrial-anchored Spire1C act as actin-nucleating proteins, which cooperate to stimulate direct actin polymerization at MERCSs, enhancing the close ER-mitochondrial contact (<30 nm), which facilitates calcium release from the ER and its uptake by mitochondria via the MCU in the IMM (Figures 5A,B) (Chakrabarti et al., 2018; Steffen and Koehler 2018). The elevated MCU-dependent intra-mitochondrial Ca^2+^ flux initiates the IMM constriction, which is primarily accomplished by CoMIC. Subsequently, mitochondrial Ca^2+^ can induce the intra-mitochondrial K+ influx via mitoBK_Ca_, leading to mitochondrial bulging and depolarization during CoMIC (Figure 5B) (Cho et al., 2017). Inhibiting the entry of mitochondrial Ca^2+^ by blocking the MCU can reduce the number of mitochondrial membrane constrictions (Chakrabarti et al., 2018). As such, enhancing the interaction between the ER and mitochondria increases the transfer of Ca^2+^ from the ER to mitochondria, which is crucial for the induction and potentiation of CoMIC. Synergistically, OMA1-mediated OPA1 cleavage can regulate CoMIC. The proteolytic processing of L-OPA1 leads to the accumulation of S-OPA1, which binds to the Mic60 subunit of the MICOS complex, preventing the outer-inner membrane tethering, and thus contributing to the IMM constriction (Figure 5B) (Cho et al., 2017). Although CoMIC does not appear to be able to completely sever the IMM, it induces the transient dissociation of the OMM–IMM contact (Figure 5B) (Cho et al., 2017; Chakrabarti et al., 2018; Hemel et al., 2021). In addition, recent studies have shown that mitochondria undergoing CoMIC have a higher probability of subsequent mitochondrial fission, furthermore, mitochondrial fission occurs at the foci of CoMIC, and conditions blocking CoMIC can also inhibit fission of mitochondria (Cho et al., 2017; Chakrabarti et al., 2018). Thus, CoMIC is a priming event that is crucial for mitochondrial fission.

Despite coupling to the OMM division, the IMM division is considered to proceed independently of DRP1 activation and to occur before full mitochondrial division (Cho et al., 2017; Chakrabarti et al., 2018). Although the IMM does not directly contact to the ER, MERCSs are indispensable to the division of IMM (Cho et al., 2017; Chakrabarti et al., 2018). As a priming event for mitochondrial fission, the foci of CoMIC is spatially specified by MERCSs. As shown in 3D images of the ER and mitochondria (labeled with GFP-Sec61b), CoMIC and subsequent mitochondrial division occur when mitochondria are surrounded by the ER (Cho et al., 2017). Moreover, observations in DN-DRP1-expressing neurons and kidney cell lines also suggest that CoMIC occurs at the ER-mitochondrial intersection (Cho et al., 2017). In addition, live imaging in control and DN- DRP1-expressing neurons showed that GFP-MFF, which marked the putative site of fission at MERCSs, was localized at the constriction site during CoMIC (Cho et al., 2017). In summary, these data indicate that the MERCSs spatially specifies the position of pre-constriction events, including INF2-mediated actin polymerization and CoMIC (Figures 5A,B).

Obviously, there are direct and extensive contacts between the ER and the outer membrane of mitochondria, where the ER twists around mitochondria to build dynamic connections. A large number of studies have illustrated that the MERCSs defines the position where the OMM division occurs (Friedman et al., 2011). Prior to the DRP1 recruitment, there is an upstream event that provides mechanical force for the formation of pre-constriction sites at MERCSs. Using platinum replica electron microscopy, a recent study found that there were dense arrays of actin filaments colocalized with mitochondrial constriction sites, and they further examined the position of non-muscle myosin II (NMII), which also has a promoting role in fission, along with INF2-mediated actin polymerization (Yang and Svitkina 2019). Moreover, it was observed that the dynamic NMII cloud existed adjacent to the constriction site, mainly along with actin fibers (Yang and Svitkina 2019). Notably, the study has proposed that NMII may ensure the constriction of actin cables, providing a pulling force to deform mitochondria and form pre-constricted sites (Yang and Svitkina 2019). Following actin−NMII-dependent constriction, DRP1 assemblies and binds to specific receptors, forming a ringlike structure at the constriction site marked by MERCSs, allowing further membrane scission (Figure 5C). Eventually, DNM2, another member of the dynamin family of GTPases, collaborates with DRP1 to perform efficient membrane fission before the fission machinery is disassembled (Figures 5D,E) (Lee et al., 2016).

### MERCSs Mark the Site of the OMM and IMM Fusion

**FIGURE 6:**
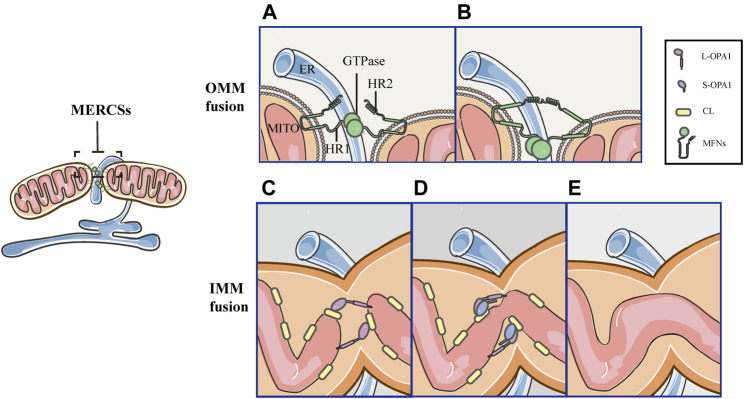
Schematic diagrams of mitochondrial fusion at MERCSs. MERCSs define the position of the OMM and IMM fusion, both of which are coordinated at the nodes of MERCSs. Mitochondrial fusion consists of five distinct steps. **(A)** OMM tethering: MFNs tether the OMM of two opposing mitochondria through their interactions in trans of the HR2 and/or GTPase domains. **(B)** OMM docking: GTP hydrolysis induces conformational changes in MFNs, which trigger docking of the OMM. Subsequently, the GTPase-dependent MFNs oligomerization completes fusion of the OMM. **(C)** L-OPA1-CL tethering: the IMM fusion begins with the interaction between L-OPA1 and CL. **(D,E)** IMM fusion: the L-OPA1-CL interaction is enhanced by S-OPA1, and the OPA1-dependent GTP hydrolysis ensures fusion of two IMMs.

Mitochondrial fusion proceeds through two successive events, MFNs-mediated OMM fusion and OPA1-driven IMM fusion (Figure 6). The initial step of the OMM fusing involves MFNs and depends on GTP-binding (Figures 6A,B). Firstly, MFNs tether the OMM of two opposite mitochondria through their trans-interactions of the HR2 and/or GTPase domains (Figure 6A). GTP hydrolysis induces dimerization of MFNs, followed by GTP-dependent MFNs oligomerization, completing the fusion of OMM (Figure 6B). As a downstream event, the tethering of L-OPA1 and CL promotes the fusion of two IMMs via GTP hydrolysis (Figures 6C–E) (Brandt et al., 2016; Ban et al., 2017; Tilokani et al., 2018). Since MFNs are involved in the tethering, docking and fusion of the OMM, their positions denote the initial point of fusion (Brandt et al., 2016; Abrisch et al., 2020). Previously, it has been reported that MERCSs recruit the mitochondrial fission machinery and mark the site of fission, however, it is unclear whether MERCSs define mitochondrial fusion sites. Recently, a new study reveals a similar coupling mechanism between MERCSs and mitochondrial fusion. It has been demonstrated that MFNs accumulate at MERCSs where fusion occurs (Abrisch et al., 2020). *In vitro* immunofluorescence staining showed that MFN1/2 puncta accumulated at the ER tubule–mitochondria intersection (Abrisch et al., 2020). Moreover, using live-cell imaging, it was strikingly found that the vast majority of fusion events were marked by MFN1 puncta (Abrisch et al., 2020). Furthermore, they confirmed the bona fide MERCSs, the close apposition between the ER and mitochondria, through an optimized dimerization-dependent fluorescent protein system (Abrisch et al., 2020). More importantly, they validated that MFN1 puncta and fusion events were localized in foci of the bona fide MERCSs (Abrisch et al., 2020). In addition, using the techniques such as fluorescence loss in photobleaching and photoconversion coupled with the conventional light microscopy, the continuity of mitochondrial compartments could be detected (Abrisch et al., 2020). As shown by Abrisch et al. (2020), the position of the IMM and OMM fusion is defined at MERCSs, and both are coordinated at the foci of MERCSs (Figure 6) (Abrisch et al., 2020).

### Mitochondrial Fission and Fusion Are Coordinated at the Same Position of MERCSs

Interestingly, both mitochondrial fission and fusion occur at MERCSs (Friedman et al., 2011; Cho et al., 2017; Kraus and Ryan 2017; Abrisch et al., 2020), whereas, whether they are coordinated at the same position or occur at separate locations of MERCSs remains unclear. This issue was examined by the same working group in two different human cell lines: U-2 OS and HeLa. Abrisch et al. (2020) observed that DRP1 and MFN1 puncta colocalized at the same position of MERCSs during both fission and fusion events, suggesting that the process of fission and fusion can spatially converge at MERCSs—both of them occur at the MERCSs, and are coordinated at the same spot—as the hotspot for mitochondrial dynamics (Abrisch et al., 2020). Consistently, bidirectional membrane dynamics occurring at MERCSs were also found in yeast, indicating that these bidirectional MERCSs nodes are broadly conserved (Abrisch et al., 2020). In conclusion, MERCSs provide a series of nodes on mitochondria where fusion and fission machineries are colocalized, and either one of them can be pushed at these sites in response to different signals (Figures 5, 6). For example, as we described above, hypoxia insults can push the reaction at MERCSs nodes toward fission, resulting in fragmented mitochondria that are beyond repair and targeted to mitophagy. Instead, other signals may guide a path to fusion, allowing damage mitochondrial to be repaired.

## FUNDC1 Regulates Mitochondrial Dynamics and Mitophagy at MERCSs Under Hypoxia

**FIGURE 7:**
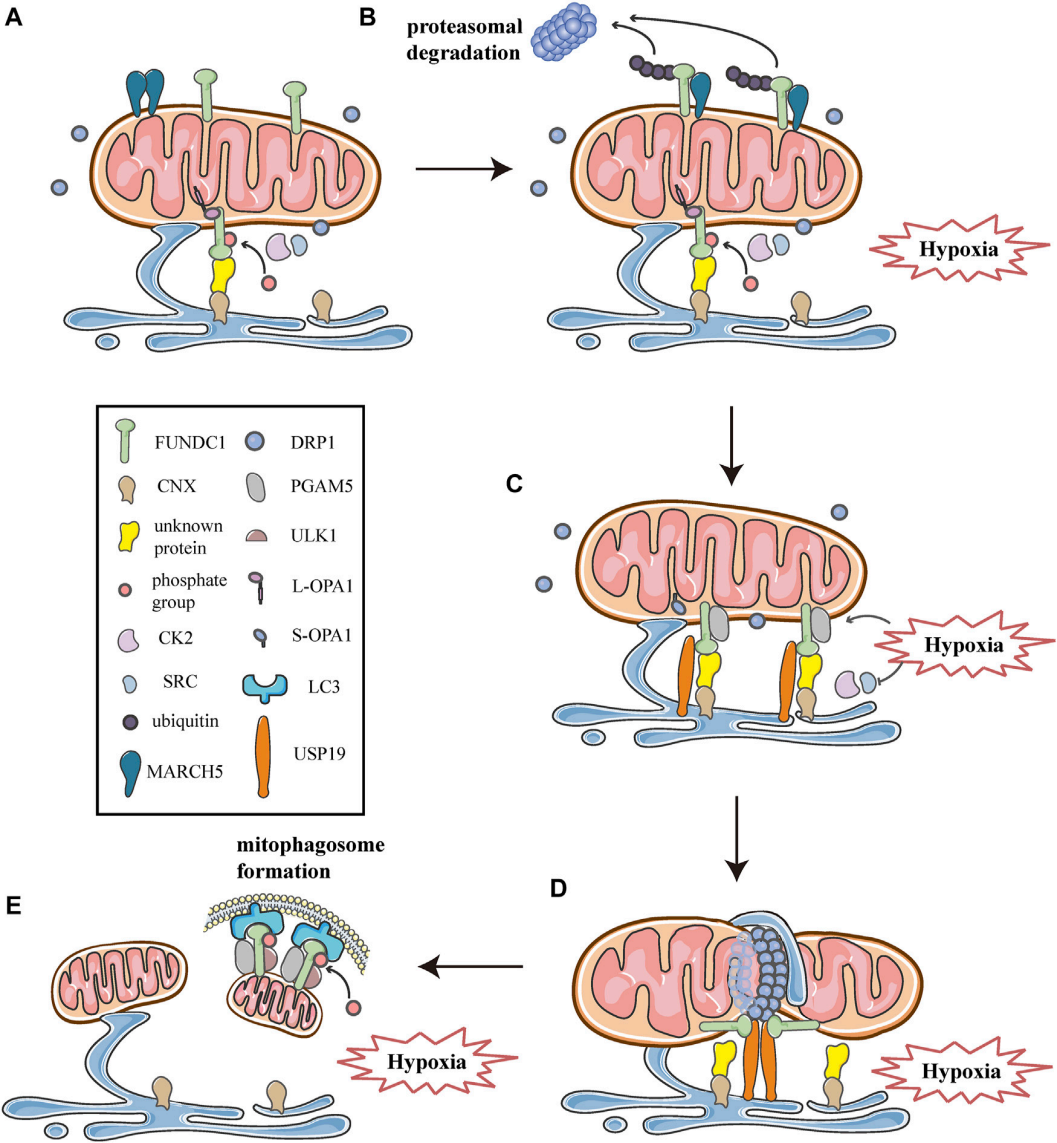
Schematic diagrams of the molecular mechanism of mitochondrial dynamics, mitophagy, and MERCSs crosstalk under hypoxia. FUNDC1 acts as a key point in the molecular machinery that regulates the close link between mitochondrial dynamics and mitophagy at MERCSs under hypoxia. **(A)** FUNDC1 is an outer membrane protein of mitochondria. Under normal conditions, only a small amount of FUNDC1 is located on MERCSs, phosphorylated by CK2 or SRC kinase, interacting with OPA1 and indirectly binding to CNX via an unknown protein. MARCH5 is a mitochondrial E3 ubiquitin ligase that localizes on the OMM, usually forming dimers or oligomers. **(B)** At the initial stage of hypoxia, the oligomer of MARCH5 disassembles, and it directly binds to FUNDC1, mediating the ubiquitin-proteasome proteolytic pathway that degrades FUNDC1. Meanwhile, the inhibitory phosphorylation by SRC and CK2 kinase suppresses the activity of FUNDC1, both of which hinder FUNDC1-mediated mitochondrial fission and mitophagy, thereby avoiding inappropriate clearance of undamaged mitochondria. **(C)** As hypoxia progresses, activities of SRC and CK2 kinase are inhibited, while PGAM5 phosphatase is activated, leading to dephosphorylation of FUNDC1 dissociated from OPA1. Moreover, USP19 significantly accumulates at MERCSs and strongly binds to FUNDC1, which is deubiquitinated and stabilized at MERCSs, where FUNDC1 indirectly interacts with CNX. The FUNDC1-CNX association contributes to the formation of a close connection between the ER and mitochondria. **(D)** Subsequently, FUNDC1 dissociates from CNX while interacting with DRP1, and USP19-mediated FUNDC1 stabilization promotes DRP1 oligomerization at MERCSs, leading to hypoxia-induced mitochondrial fission. **(E)** At the late stage of hypoxia, FUNDC1 binds to LC3 and forms an mitophagosome, which engulfs and selectively removes impaired mitochondria. Meanwhile, the upstream ULK1 complex phosphorylates FUNDC1 to promote the process of mitophagy.

As described above, multiple processes of mitochondrial dynamics are collectively regulated at MERCSs. Moreover, previous researches have shown that the site of MERCSs is also significant for mitophagosome formation during mitophagy (Böckler and Westermann 2014). Given the critical role of MERCSs in these processes, the current study suggests that it serves as a platform for some key molecules that can synergistically modulate mitochondrial dynamics and mitophagy. In addition to acting as a receptor that recruits DRP1 and mediates mitophagy, FUNDC1 is also a component of the MERCSs fraction in cells under hypoxia (Wu et al., 2016a; Wu et al., 2016b). Further investigations have revealed the mechanisms involved in the functioning of FUNDC1 at the spot of MERCSs to integrate mitochondrial fission and subsequent mitophagy under hypoxic conditions. As observed in HeLa cells, they found that under hypoxic conditions, the number of MERCSs increased and FUNDC1 significantly accumulated at MERCSs, whereas under normoxic conditions, only a small amount of phosphorylated FUNDC1 was localized to MERCSs, interacting with OPA1 (Figure 7A) (Wu et al., 2016a; Wu et al., 2016b). The accumulation of FUNDC1 at MERCSs is supported by the interaction with calnexin (CNX), an ER membrane protein, which is mediated by an unknown intermediate protein (Figure 7A) (Wu et al., 2016b). And the knockdown of CNX considerably reduces the amount of FUNDC1 at MERCSs (Wu et al., 2016b). Interestingly, the same region of FUNDC1 can interact with DRP1 and CNX in a time-dependent manner during hypoxia. FUNDC1 is dephosphorylated by PGAM5 and dissociates from OPA1, and then rapidly binds to CNX via an unknown intermediate protein, promoting its localization to MERCSs under hypoxia (Figure 7C) (Wu et al., 2016b). Correspondingly, the association of FUNDC1-CNX promotes to form close connections between the ER and mitochondria (Wu et al., 2016b). When cells are subjected to prolonged hypoxia, FUNDC1 dissociates from CNX, while preferentially interacts with DRP1, driving the initiation of mitochondrial fission (Figure 7D), which separates dysfunctional mitochondria and produces mitochondrial fragments with appropriate sizes for degradation by mitophagy (Wu et al., 2016a). At the late stage of hypoxia, FUNDC1 directly binds to LC3-II, which is abundantly produced under hypoxia, and recruits LC3-bound isolation membranes that expand around the mitochondria, forming mitophagosomes to engulf small pieces of damage mitochondria (Figure 7E) (Liu et al., 2012). Eventually, mitophagosomes are trafficked to lysosomes for degradation (Liu et al., 2014; Liu et al., 2012). Additionally, general autophagy machineries are activated by the upstream ULK1 complex, which can phosphorylate FUNDC1 at Ser17, promoting the course of mitophagy (Figure 7E) (Wu et al., 2014). In summary, FUNDC1, CNX, and DRP1 cooperate at MERCSs, coupling mitochondrial dynamics to mitophagy in response to hypoxia (Figure 7).

However, a recent study has proposed that, as one of the early events in response to hypoxia, FUNDC1 undergoes ubiquitin-mediated degradation in a proteasome-dependent manner (Figure 7B) (Chen et al., 2017a; Chen et al., 2017b). As shown by western blotting and co-immunoprecipitation in Hela cells exposed to hypoxia for different times, FUNDC1 degradation occurs prior to its dephosphorylation and activation, both of which are essential for subsequent mitochondrial fission and mitophagy (Chen et al., 2017a). Furthermore, the group has demonstrated that MARCH5, a mitochondrial E3 ubiquitin ligase located on the OMM, is responsible for the ubiquitylation of FUNDC1 (Chen et al., 2017a). Previous researches have shown that MARCH5 can form dimers or oligomers (Figure 7A), which is necessary for the clearance of its own dysfunctional mutant to maintain mitochondrial quality (Kim et al., 2016). Recently, Chen et al. have found that the self-interaction of MARCH5 dramatically reduces during hypoxia, as shown by cross-linking analysis, while the interaction between MARCH5 and FUNDC1 is progressively enhanced (Figure 7B) (Chen et al., 2017a). Furthermore, using *in vivo* and *in vitro* ubiquitination assays, they have demonstrated that MARCH5 mediates FUNDC1 ubiquitylation at lysine119 (K119), promoting the degradation of FUNDC1 (Figure 7B), whereas MARCH5 ablation leads to the accumulation of FUNDC1 and enhanced mitophagy (Chen et al., 2017a). Indeed, the MARCH5/FUNDC1 axis, as a negative feedback mechanism, fine-tunes mitophagy in response to hypoxia stress, avoiding improper removal of cellular mitochondria (Chen et al., 2017a; Chen et al., 2017b). However, when cells are exposed to prolonged and/or severe hypoxia stress, damaged or unwanted mitochondria increasingly accumulate and should be removed by mitophagy for cell health (Chen et al., 2017a; Chen et al., 2017b). Paradoxically, MARCH5 mediated ubiquitination and degradation of FUNDC1 appear to impede the progression of subsequent fission and mitophagy.

More recently, a novel investigation has suggested a molecular mechanism that promotes the stabilization and accumulation of FUNDC1 at MERCSs, thus contributing to mitochondrial fission and subsequent mitophagy. Using percoll density-gradient centrifugation in HeLa cells, Chai et al. have found that FUNDC1 significantly accumulates at MERCSs under hypoxia, which is consistent with previous studies (Wu et al., 2016b; Chai et al., 2021). More importantly, they have reported that ubiquitin-specific protease 19 (USP19), a deubiquitinase located on the ER, also markedly accumulates at MERCSs due to hypoxic treatments (Figure 7C), further verified by immunoelectron microscopy (Chai et al., 2021). Furthermore, using immunoprecipitation coupled with pull-down assays, the investigation has confirmed that USP19 directly binds to FUNDC1, and that the interaction is strongly enhanced under hypoxic conditions (Figure 7C) (Chai et al., 2021). The interaction between USP19 and its substrate FUNDC1 is also verified by co-immunoprecipitation assays and glutathione S- transferase (GST) affinity isolation assays (Chai et al., 2021). Moreover, *in vitro* deubiquitination assays and immunoprecipitation assays proved that under hypoxia, USP19 stabilized FUNDC1 at MERCSs through deubiquitination of FUNDC1 at K119, which was further confirmed by immunoelectron microscopy in USP19 knockout cells (Chai et al., 2021). Whereas the hypoxia-induced FUNDC1 accumulation at MERCSs was prevented in USP19 knockout HeLa cells, as shown by immunoelectron microscopy (Chai et al., 2021). In addition, the USP19-mediated stabilization of FUNDC1 at MERCSs is crucial for DRP1 oligomerization and hypoxia-induced mitochondrial fragmentation (Figure 7D) (Chai et al., 2021). Interestingly, Chai et al. also found that USP19 could interact with DRP1, although the exact mechanism is still unknown, and whether USP19 promotes the oligomeric transfer of DRP1 to MERCSs requires further investigation (Chai et al., 2021). Collectively, in response to hypoxia, USP19 markedly accumulates at MERCSs and strongly interacts with FUNDC1, mediating its deubiquitination at K119, which promotes the localization of FUNDC1 to MERCSs and subsequent DRP1 oligomerization, thereby inducing mitochondrial fission (Figures 7C,D) (Chai et al., 2021). In addition, the research proposes the notion that USP19, FUNDC1, and DRP1 act as a complex that synergistically mediates mitochondrial fission at the MERCSs under hypoxia (Figure 7D) (Chai et al., 2021). Interestingly, USP19 and MARCH5 can associate with FUNDC1 at the same residue K119, mediating the deubiquitination and ubiquitination of FUNDC1, respectively (Chen et al., 2017a; Chai et al., 2021). It appears that there is a competitive relationship between USP19 and MARCH5, both of which may reversely regulate the deubiquitination/ubiquitination of FUNDC1 at K119 by competitively binding to FUNDC1 under hypoxic conditions (Chen et al., 2017a; Chai et al., 2021). Initial hypoxia stress induces disassembly of MARCH5 oligomers and enhances the interaction between MARCH5 and FUNDC1, which mediates the ubiquitination degradation of FUNDC1, thus circumventing improper removal of mitochondria (Chen et al., 2017a; Chai et al., 2021). As hypoxia progresses, FUNDC1 may preferably bind to USP19, which deubiquitinates and stabilizes FUNDC1 at MERCSs, as a result, mitochondrial fission is initiated, and mitophagy is promoted to eliminate damage mitochondria (Figure 7D) (Chai et al., 2021).

Under hypoxic conditions, FUNDC1 acts as a key point in the molecular machinery that regulates the intimate connection between mitochondrial dynamics and mitophagy at the site of MERCSs. Recently, Wu et al. have demonstrated the critical role of FUNDC1 in the regulation of MERCSs, mitochondrial dynamics and mitochondrial functions in cardiomyocytes and in intact hearts (Wu et al., 2017). The group found that FUNDC1 was localized to MERCSs, consistent with previous studies, and that the ablation of FUNDC1 in mouse cardiomyocytes disrupted MERCSs, elongated mitochondria and compromised mitochondrial functions (Wu et al., 2016a; Wu et al., 2016b; Wu et al., 2017). Consistently, mice with cardiomyocyte-specific FUNDC1 gene knockout exhibited cardiac dysfunction, which was further exacerbated by ischemia and hypoxia (Wu et al., 2017). More importantly, the research showed that levels of FUNDC1 were significantly lower in patients with heart failure compared to control donors, meanwhile, contacts between the ER and mitochondria were reduced and mitochondria were more elongated in heart failure hearts (Wu et al., 2017). Another study identifies a new molecule that is involved in the regulation of mitochondrial dynamics under hypoxia. The group has reported that filamin A (FLNa), an actin-binding protein, functionally couples DRP1 to actin at the fission site marked by MERCSs, and activates DRP1 through its GTPase activity during hypoxia in rat cardiomyocytes (Nishimura et al., 2018). The DRP1-FLNa interaction leads to mitochondrial hyperfission, which is associated with myocardial senescence and heart failure after myocardial infarction (MI), whereas inhibition of the DRP1-FLNa complex formation can suppress hypoxia-mediated mitochondrial fragmentation and attenuate the progression of heart failure after MI (Nishimura et al., 2018). However, how the DRP1-FLNa interaction is regulated under hypoxia is still unclear. Whether other DRP1 binding proteins, such as FUNDC1 and actin, are involved in regulating this interaction remains to be further investigated.

## Conclusion

Mitochondria, as major energy-producing organelles, are highly sensitive to hypoxia stress and respond dynamically under hypoxia, which can minimize ROS formation and reduce the risk of cellular demise and tissue damage. The quality control mechanism is critical for the maintenance of mitochondrial functions. In cells subjected to hypoxia, these well-orchestrated processes may serve as part of the mitochondrial response program to hypoxia, including the commitment of mitochondrial dynamics and the involvement of mitophagy. The balance of mitochondrial fission and fusion events is critical not only for maintaining mitochondrial morphology, but also for isolating dysfunctional mitochondria and their rapid repair. Indeed, mitochondrial fission observed during hypoxia can be considered as a preventive mechanism for the exit of damaged mitochondria from mitophagy, which is necessary for a health mitochondrial network. Notably, mitochondrial dynamics and mitophagy are coupled at MERCSs in response to hypoxia, and the machineries of these well-organized processes spatially converge at the specific spot of MERCSs. The cooperation of mitochondrial dynamics, mitophagy and MERCSs is really important for the adaptive response of mitochondria to hypoxia, which may provide novel insights into hypoxia-induced diseases. However, since molecules involved in the crosstalk process have not been fully identified, new techniques and methods may be needed to discover new molecules that mediate these biological processes at the MERCSs. And different human diseases that related to the mechanism need to be further explored.
